# Clinical Performance and Survival of Bulk-Fill Resin Composite Posterior Restorations in Primary Teeth: A Systematic Review and Meta-Analysis

**DOI:** 10.3390/jcm15020415

**Published:** 2026-01-06

**Authors:** Samille Biasi Miranda, Rodrigo Barros Esteves Lins, Matheus de Farias Queiroz, Caroline de Farias Charamba Leal, Giovana Lordsleem de Mendonça, Tayana de Albuquerque Dias, Marcos Antonio Japiassú Resende Montes

**Affiliations:** 1 Department of Dental Materials, Faculty of Dentistry, University of Pernambuco, Recife 50100-130, PE, Brazil; samille.biasi@upe.br (S.B.M.); matheus.fariasqueiroz@upe.br (M.d.F.Q.); caroline.charamba@upe.br (C.d.F.C.L.); giovana.lordsleem@upe.br (G.L.d.M.); marcos.japiassu@upe.br (M.A.J.R.M.); 2 School of Dentistry, Federal University of Alagoas, Maceió 57072-900, AL, Brazil; tayana.dias@foufal.ufal.br

**Keywords:** bulk-fill resin composite, primary teeth, glass ionomer cements, pediatric dentistry, randomized controlled trials, systematic review

## Abstract

**Background/Objectives**: This systematic review and meta-analysis aimed to evaluate the clinical performance and survival of bulk-fill resin composite restorations in posterior primary teeth, compared with conventional resin composites and glass ionomer cements (GICs). **Methods**: The protocol was registered in PROSPERO (CRD42024539097) and conducted according to PRISMA guidelines. Electronic searches were performed in PubMed, Embase, Web of Science, Scopus, and Cochrane databases up to December 2025. Randomized clinical trials (RCTs) evaluating bulk-fill resin composite restorations in primary molars were included. Restoration survival was considered the primary clinical endpoint. Secondary outcomes included marginal integrity, marginal discoloration, color match, postoperative sensitivity, secondary caries, retention, and anatomical form, assessed using USPHS or FDI criteria. Meta-analyses were performed for color match, marginal discoloration, and marginal integrity using a random-effects model. Comparisons involving GICs were synthesized descriptively. Risk of bias was assessed using the RoB 2 tool, and certainty of evidence was evaluated using GRADE. **Results**: Six RCTs involving 1220 restorations in children aged 3 to 10 years were included, with follow-up periods ranging from 6 to 24 months. Survival rates were high across all materials. Meta-analyses up to 12 months showed no significant differences between bulk-fill and conventional resin composites for color match, marginal discoloration, or marginal integrity. Most RCTs were judged as having low risk of bias, with one study presenting some concerns. The certainty of evidence was rated as moderate, mainly due to imprecision related to sample size and limited reporting of confidence intervals. **Conclusions**: Bulk-fill resin composites demonstrate clinical performance and survival comparable to conventional resin composites in posterior restorations of primary teeth over follow-ups of up to 12 months. Based on RCTs with predominantly low risk of bias and moderate certainty of evidence, bulk-fill composites represent a reliable and efficient restorative option in pediatric dentistry.

## 1. Introduction

Restorative materials that simplify clinical procedures and reduce treatment time are increasingly sought after, particularly for the management of large posterior cavities in primary teeth [1]. Conventional resin composites require incremental layering techniques to reduce polymerization shrinkage stress, which increases chair time and may compromise clinical outcomes, especially in pediatric patients with limited cooperation [2]. Moreover, previous evidence has shown that although resin composites present favorable mechanical properties for use in pediatric dentistry, their clinical success in primary teeth is highly dependent on adequate polymerization, marginal integrity, and operator-related factors, particularly when incremental techniques are used [3]. To address these limitations, bulk-fill resin composites were developed to allow placement in thicker increments—up to 4–5 mm—while maintaining adequate polymerization and acceptable mechanical properties [4].

In primary dentition, glass ionomer cements (GICs) and resin composites remain the most commonly used restorative materials, with resin composites generally showing lower failure rates [2]. However, the technique sensitivity and longer operative time associated with conventional incremental composites may negatively affect treatment efficiency and patient comfort [5]. Bulk-fill composites have therefore been proposed as a promising alternative, as they may reduce clinical time and simplify restorative procedures while maintaining satisfactory marginal integrity and clinical longevity [6,7]. Advances in bulk-fill resin composites have improved depth of cure and reduced polymerization shrinkage stress without compromising mechanical performance or biocompatibility [6].

Bulk-fill composites are well established in permanent dentition, with several clinical studies reporting favorable medium and long-term outcomes [8,9]. Their use in primary teeth, however, has received comparatively less attention. This distinction is clinically relevant, as primary teeth present specific anatomical and structural characteristics, such as thinner enamel and dentin and reduced mineralization, that may influence adhesive performance, marginal adaptation, and restoration survival [1,2]. In addition, current clinical guidelines in pediatric dentistry emphasize minimally invasive and efficient treatment strategies, reinforcing the need for restorative materials that combine ease of use with reliable clinical effectiveness [10,11].

Previous systematic reviews have assessed bulk-fill resin composites; however, most focused on permanent dentition or combined data from permanent and primary teeth. Evidence specifically related to primary teeth remains limited, with few randomized clinical trials and heterogeneous outcome definitions, which hampers quantitative synthesis. In addition, recently published RCTs in primary molars and the consistent use of standardized clinical evaluation systems, such as the USPHS and FDI criteria, have not been fully incorporated into earlier reviews.

Therefore, this systematic review and meta-analysis aimed to evaluate the clinical performance and survival of bulk-fill resin composite restorations in posterior primary teeth, compared with conventional resin composites and glass ionomer cements. Restoration survival was defined as the primary outcome, while marginal integrity, marginal discoloration, color match, secondary caries, retention, postoperative sensitivity, and anatomical form were considered secondary outcomes. The review was guided by the hypothesis that bulk-fill resin composites present clinical performance and survival comparable to those of conventional resin composites and glass ionomer cements in primary teeth, while also considering the possibility of superior performance of bulk-fill materials based on the synthesis of available evidence.

## 2. Materials and Methods

### 2.1. Protocol and Registration

This systematic review was conducted following the guidelines of the PRISMA (Preferred Reporting Items for Systematic Reviews and Meta-Analyses) guidelines (Supplementary Material Table S1) [12]. Prior to commencement, the methodology of this study was registered in PROSPERO (International Prospective Register of Systematic Reviews) with the protocol number #CRD42024539097.

### 2.2. Eligibility Criteria

The guiding question for this review was “Does Bulk-Fill RBC exhibit clinical performance similar to conventional RBC or glass ionomer cement in posterior restorations of primary teeth? The Population, Intervention, Comparison, and Outcome of the study were guided by the PICOS strategy. The Population/participants (P) consisted of patients with posterior restorations in deciduous teeth. The Intervention (I) analyzed was direct restoration with Bulk-Fill RBC, and the Comparator (C) was Conventional RBC (incremental technique). The Outcomes (O) evaluated were color match, marginal discoloration, marginal integrity, post-operative sensitivity, secondary caries, retention, and anatomical form. The study design (S) was a Randomized Clinical Trial.

Inclusion criteria for the studies were: (1) randomized clinical trials (2) evaluate clinical performance of Bulk Fill resin composite in posterior restorations; (3) Studies that used the United States Public Health Service (USPHS) Criteria or World Dental Federation (FDI) criteria to evaluate clinical performance of the resin composites; (4) Studies that used conventional resin composites or Glass Ionomer Cement as control; (5) Studies with only primary teeth. Exclusion criteria: (1) Studies evaluating clinical performance with methods other than those outlined in the predefined eligibility criteria (e.g., studies that do not report outcomes using USPHS or FDI criteria); (2) In vitro studies; (3) Studies that included experimental Bulk-Fill RBC; (4) Unpublished information in the scientific literature (e.g., study protocols, abstracts, or non-peer-reviewed reports); (5) Studies with unavailable full text.

### 2.3. Information Sources and Search Strategy

PubMed, Embase, Web of Science, Scopus, and Cochrane were accessed in December 2025, using a search strategy that did not limit a chronological period to find clinical studies evaluating the clinical performance of Bulk-Fill RBCs in direct restorations in primary teeth. The following terms were used across the databases: [(“Bulk Fill” OR “Bulkfill” OR “Bulk-Fill” OR “Bulk Filled” OR “Bulk Filling”) AND (“Permanent Dental Restorations” OR “Permanent Dental Restoration” OR “Dental Permanent Fillings” OR “Dental Permanent Filling”) AND (“Primary Teeth” OR “Deciduous Tooth” OR “Primary Tooth” OR “Deciduous Teeth” OR “Primary Dentition” OR “Primary Dentitions” OR “Milk Teeth” OR “Milk Tooth” OR “Deciduous Dentition” OR “Deciduous Dentitions”)] (Table 1). In addition to the electronic database searches, trial registries, gray literature sources, and the reference lists of relevant reviews and included studies were manually screened to identify additional eligible studies.

### 2.4. Selection Process

Studies were saved and systematically organized using the online program (Rayyan, Qatar Computing Research Institute) [13]. Duplicates were first removed, and then titles and abstracts were read to determine whether the studies met the predefined criteria. The selection process was conducted in a blinded and independent manner by two authors (S.B.M. and M.d.F.Q.), previously calibrated, and discrepancies were discussed with a third author (M.A.J.R.M.). The calibration process prior to study selection, data extraction, and risk of bias assessment involved joint reading and assessment of 10 articles to ensure congruence in interpreting and applying the eligibility criteria. This collaborative review fostered a unified approach to article selection based on predefined criteria. Any disparities encountered during independent selection were resolved through discussion and consensus with the third author (M.A.J.R.M.). Formal inter-reviewer agreement was calculated only for the final study selection stage using the Kappa score [14]. For data extraction and risk of bias assessment, consistency between reviewers was ensured through iterative consensus discussions. Eligible articles underwent thorough reading, and their data were meticulously extracted. Mendeley software v2.141.0 was used as a reference manager (Elsevier, Mendeley).

### 2.5. Data Collection Process

Two authors (S.B.M. and M.d.F.Q.), previously calibrated, performed data extraction using a guiding table covering the main methodological characteristics of the studies. Key data included author/year, Study Design, number of subjects and age range, number of restored teeth, rubber dam isolation, Depth of carious lesions, follow-up period, RBCs used, type of cavity, Adhesive system, light curing unit, and analysis criteria.

### 2.6. Study Risk of Bias Assessment

The included studies underwent a risk of bias evaluation conducted by two authors (S.B.M. and M.d.F.Q.), who had been previously calibrated. The assessment utilized the Cochrane Risk of Bias for Randomized Trials version 2 (RoB 2) tool [15], which comprises domains assessing bias related to the randomization process, deviations from intended interventions, missing outcome data, measurement of outcomes, and selection of reported results. Each domain is accompanied by signaling questions designed to systematically extract relevant information for bias assessment, with responses categorized as yes, probably yes, probably no, no, or no information. Following the completion of signaling questions, a risk-of-bias judgment is made, categorized as low risk of bias, some concerns, or high risk of bias. The RoB 2 tool incorporates algorithms that connect responses to signaling questions with suggested risk-of-bias assessments for each domain. In instances of disagreement between the two assessors, a third assessor was consulted to achieve consensus (M.A.J.R.M.).

### 2.7. Effect Measures and Synthesis Methods

Meta-analysis was performed using a random-effects model. Review Manager version 5.4 (The Cochrane Collaboration, Copenhagen, Denmark) was used to calculate the risk difference with a 95% confidence interval. For this analysis, data were dichotomized. Acceptable restorations were defined as those receiving Alpha or Bravo scores according to the USPHS criteria and scores 1, 2, or 3 according to the FDI criteria. Unacceptable restorations were defined as those receiving Charlie scores under the USPHS criteria or scores 4 and 5 according to the FDI criteria. Only unacceptable scores, representing clinically relevant failures, were included in the meta-analysis. Analyses were conducted using two subgroups based on follow-up duration: baseline (1 to 7 days) and 12 months. This dichotomization was adopted to harmonize outcomes across different clinical evaluation systems; however, it does not fully capture intermediate clinical changes (e.g., USPHS Bravo or FDI scores 2–3) and may reduce sensitivity to detect moderate differences between restorative materials.

Meta-analyses were restricted to color match, marginal discoloration, and marginal integrity because these outcomes were consistently reported across the included randomized clinical trials using comparable USPHS or FDI criteria and similar follow-up periods. Other clinically relevant outcomes, such as secondary caries, fractures, and retention, were reported heterogeneously and were therefore synthesized descriptively.

### 2.8. Certainty Assessment

The certainty of evidence for each outcome was evaluated utilizing the Grading of Recommendations, Assessment, Development, and Evaluation (GRADE) tool [16], accessible at http://www.gradeworkinggroup.org/, accessed on 10 August 2025. This tool evaluates the study design and considers factors such as risk of bias, imprecision, inconsistency, indirectness of evidence, and publication bias to potentially determine the quality of evidence. Each aspect is appraised as having “no limitation,” “serious limitations,” or “very serious limitations,” allowing for classification of evidence quality as high, moderate, low, or very low. Lower quality indicates that the estimate may deviate significantly from the actual effect.

## 3. Results

### 3.1. Study Selection

**Figure 1:**
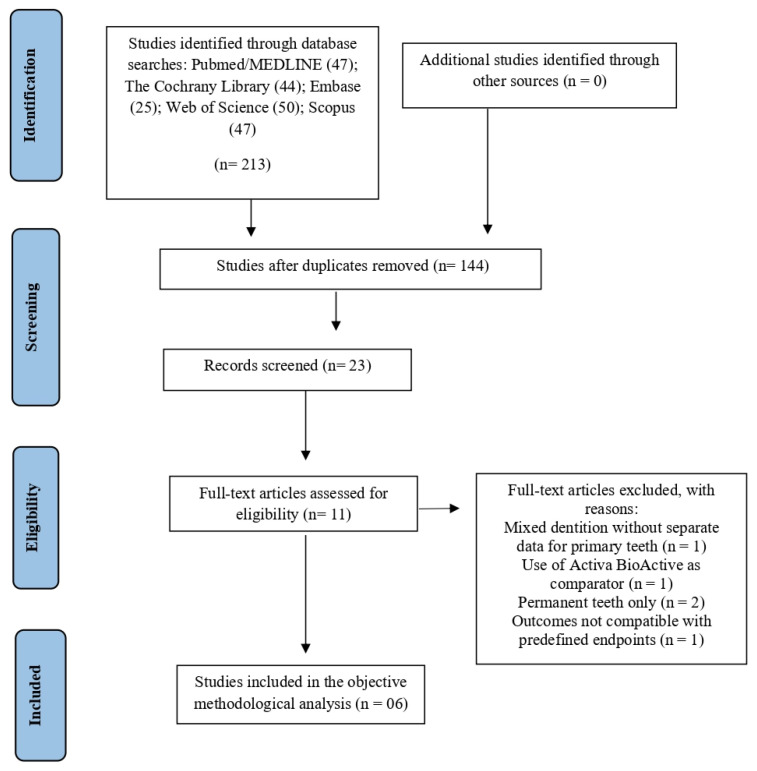
PRISMA flow diagram of the literature search and selection criteria.

A total of 213 records were identified through database searches conducted in December 2025, including PubMed/MEDLINE (47), Embase (25), Web of Science (50), The Cochrane Library (44), and Scopus (50). No additional eligible studies were identified through searches of trial registries, gray literature sources, or screening of reference lists. After removing duplicates, 144 records remained for screening. Based on title and abstract analysis, 133 articles were excluded for not meeting the predefined inclusion criteria, resulting in 11 articles selected for full-text assessment. Following the full-text evaluation, five studies were excluded, and the reasons for full-text exclusions are presented in Supplementary Table S2. Therefore, a total of six studies were included in the final methodological analysis (Figure 1).

The results of the inter-examiner agreement test showed an “almost perfect agreement” between the examiners in the article selection phase. The indices of the databases were PubMed/Medline (1.0), Embase (0.83), The Cochrane Library (0.8), Web of Science (1.0), and Scopus (1.0).

### 3.2. Study Characteristics

The characteristics of the studies are observed in Table 2.

The included randomized clinical trials [17,18,19,20,21,22], published between 2019 and 2022, evaluated pediatric patients aged 3 to 10 years, with sample sizes ranging from 27 to 80 children and 54 to 160 restored primary molars. Follow-up periods varied from 6 to 24 months. Sarapultseva and Sarapultsev (2019) [19] enrolled 27 children (3–6 years), Akman and Tosun (2020) [17] included 30 participants (6–10 years), Ehlers et al. (2019) [18] reported a mean age of 6.7 ± 1.2 years in 32 children, and Massa et al. (2022) [22] evaluated 62 children (mean age 5.9 ± 1.7 years). Gindri et al. (2022) [21] and Oter et al. (2019) [20] enrolled 65 and 80 children, respectively. All studies were conducted in a clinical setting, assessing Class I or II cavities in primary molars. Isolation methods included a rubber dam [21,22] or cotton rolls with suction [17,20].

Bulk-fill resin composites were compared to conventional resin composites or resin-modified glass ionomer cements. The bulk-fill materials included Filtek Bulk Fill (four studies), Venus Bulk Fill, SonicFill, X-tra fil, and SDR. Controls were restored with Filtek Z250, Filtek Z350 XT, Ceram X, or Dyract eXtra; one study also tested Vitremer. Adhesive protocols mostly involved universal adhesives, such as Scotchbond Universal, in self-etch or selective-etch mode. Other systems included Clearfil SE Bond and Prime and Bond NT. Light-curing units varied (e.g., Elipar S10, Valo LED), with exposure times between 10 and 20 s per increment. Clinical performance was evaluated using either the FDI or modified USPHS criteria, depending on the trial.

### 3.3. Clinical Performance of Restorations

Across all follow-up periods, a total of 1220 restorations were evaluated (Incremental RBC: n = 451; GIC: n = 138; Bulk-fill RBC: n = 631). At 3 and 6 months, the overall failure rate was <2%, with no clinically relevant changes observed in most groups. The only recorded events were one case of loss of retention in the bulk-fill RBC group (1/160; 0.6%) [20] and minor marginal integrity changes in two restorations (2/70; 2.9%) [21]. At 12 months, restoration survival remained high (>95%). Failures were infrequent and included marginal integrity loss (Incremental RBC: 2/70; 2.9%; Bulk-fill RBC: 5/70; 7.1%), fractures (Incremental RBC: 2/70; 2.9%; Bulk-fill RBC: 1/70; 1.4%), secondary caries (Incremental RBC: 1/70; 1.4%; Bulk-fill RBC: 1/70; 1.4%), and anatomic form changes in the GIC group (2/70; 2.9%). At 18 months, Sarapultseva and Sarapultseva (2019) [19] reported no failures among the evaluated restorations (0/54; 0%), whereas Massa et al. (2022) [22] observed higher failure frequencies, particularly in the GIC group (marginal discoloration: 5/72; 6.9%; secondary caries: 4/72; 5.6%; fractures: 7/72; 9.7%; anatomic form: 4/72; 5.6%) and in the bulk-fill RBC group (marginal discoloration: 2/72; 2.8%; marginal integrity: 5/72; 6.9%; secondary caries: 2/72; 2.8%; fractures: 10/72; 13.9%; anatomic form: 2/72; 2.8%). At 24 months, only one study reported data [18], showing 100% survival in all groups (0 failures among 54 restorations). Detailed absolute numbers and corresponding percentages for all clinically relevant outcomes and follow-up periods are presented in Table 3.

### 3.4. Risk of Bias in Studies

**Figure 2:**
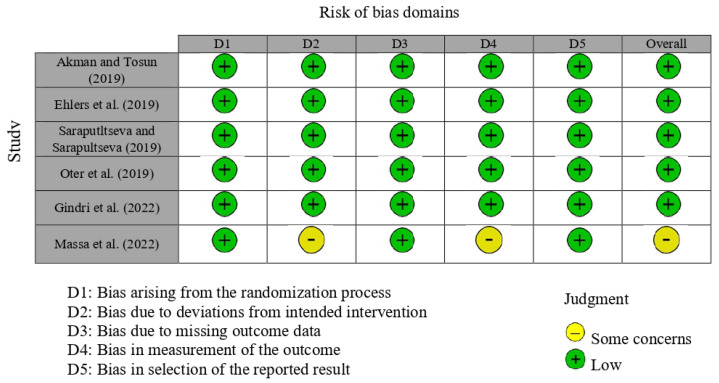
Risk of bias analysis for the randomized clinical trials [17,18,19,20,21,22].

The RoB 2 tool was applied to assess the risk of bias in the included randomized clinical trials. Overall, most studies [17,18,19,20] were judged to present a low risk of bias across the majority of the five domains, including the randomization process (D1), deviations from intended interventions (D2), missing outcome data (D3), measurement of the outcome (D4), and selection of the reported result (D5). However, some concerns were identified, mainly related to deviations from intended interventions and measurement of outcomes. In particular, Massa et al. (2022) [22] presented some concerns due to the lack of blinding of both operators and outcome assessors, which may have influenced the application of the intervention and the evaluation of subjective clinical criteria. When considering the body of evidence as a whole, these limitations were taken into account in the GRADE assessment and contributed to rating the certainty of evidence as moderate (Figure 2). When considering the body of evidence as a whole, these limitations were incorporated into the GRADE assessment and contributed to downgrading the certainty of evidence to moderate (Figure 2).

### 3.5. Results of Syntheses

For the meta-analysis, the studies that applied the United States Public Health Service (USPHS) criteria were extracted for the proportion of restorations considered with worse scores (“Charlie”), and studies that used the World Dental Federation (FDI) criteria were extracted for the equivalent worse scores (scores 4 and 5). The following clinical parameters were analyzed: color match, marginal discoloration, and marginal integrity.

**Figure 3:**
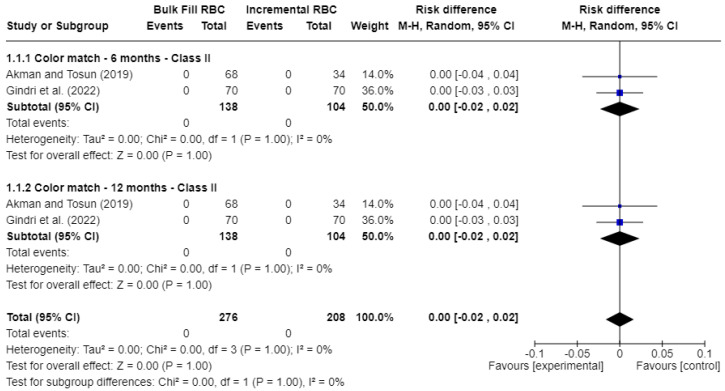
Forest plot of color match [17,21].

Figure 3 presents the results for color match. Two studies were included. No statistically significant differences (*p* > 0.05) were found between bulk-fill and conventional resin composites. The risk difference (RD) was 0.00 at 6 months (95% CI: −0.02 to 0.02; *p* = 1.00; I^2^ = 0%) and also 0.00 at 12 months (95% CI: −0.02 to 0.02; *p* = 1.00; I^2^ = 0%).

**Figure 4:**
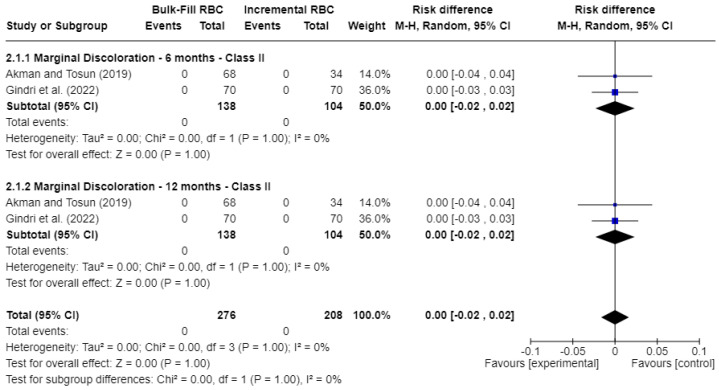
Forest plot of marginal discoloration [17,21].

Figure 4 shows the outcomes for marginal discoloration. Similarly, no statistically significant differences (*p* > 0.05) were observed. The risk difference (RD) was 0.00 at both 6 months (95% CI: −0.02 to 0.02; *p* = 1.00; I^2^ = 0%) and 12 months (95% CI: −0.02 to 0.02; *p* = 1.00; I^2^ = 0%).

**Figure 5:**
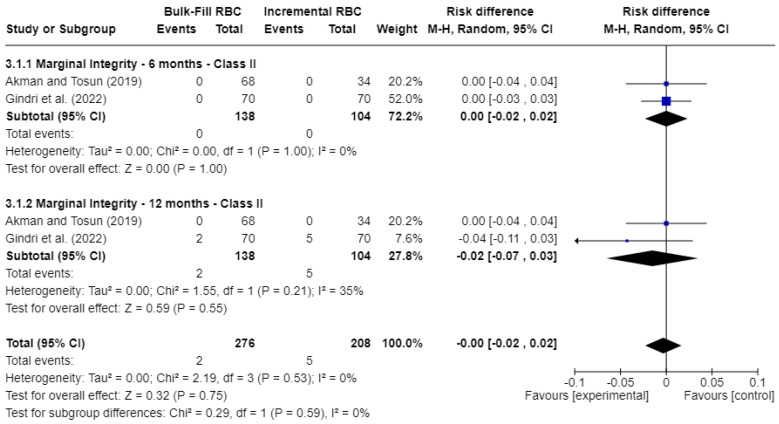
Forest plot of marginal integrity [17,21].

Figure 5 displays the analysis of marginal integrity. At 6 months, the risk difference (RD) was 0.00 (95% CI: −0.02 to 0.02; *p* = 0.75; I^2^ = 0%), indicating no significant difference between the groups. At 12 months, the risk difference (RD) was −0.02 (95% CI: −0.07 to 0.03; *p* = 0.55), still not statistically significant, though moderate heterogeneity was observed (I^2^ = 35%).

Overall, the meta-analyses showed no statistically significant differences between bulk-fill and conventional resin composites for color match, marginal discoloration, and marginal integrity at baseline and 12 months. However, most pooled comparisons were based on a very low number of failure events, resulting in risk differences centered close to 0.00 with wide confidence intervals, as shown in Figure 3, Figure 4 and Figure 5. These findings reflect limited statistical power to detect small or moderate differences between materials.

### 3.6. Certainty of Evidence

The quality of evidence for the clinical outcomes evaluated was rated as moderate according to the GRADE approach (Table 4). Three randomized controlled trials (RCTs) were included for each outcome: marginal discoloration, marginal integrity, and color match. For all outcomes, risk of bias, inconsistency, and indirectness were judged as not serious, as the studies presented appropriate methodologies, consistent findings, and direct applicability to clinical practice. Imprecision was considered serious, mainly due to the limited number of RCTs, relatively small sample sizes, and the presence of wide or unreported confidence intervals, which reduced the precision of the effect estimates. Consequently, the certainty of evidence was downgraded by one level, resulting in a moderate level of confidence for all three outcomes.

## 4. Discussion

Bulk-fill composites have shown favorable clinical performance in pediatric dentistry, particularly in primary teeth. Their ability to simplify the restorative process by allowing thicker increments is crucial for improving treatment efficiency, especially in pediatric patients with limited cooperation [1,21]. Meta-analyses showed no statistically significant differences in marginal integrity, marginal discoloration, or color match between groups. However, these findings should be interpreted with caution, as most pooled comparisons were based on a very low number of failure events, resulting in wide confidence intervals and limited statistical power to detect moderate differences between materials. Comparisons with glass ionomer cement were reported qualitatively but not included in the meta-analyses.

In all included studies, survival rates were high, and failures related to marginal integrity, marginal discoloration, or secondary caries were rare. However, these findings should be interpreted with caution due to the low event rates and small sample sizes, which limit the reliability of the results. While the null hypothesis is accepted, the strength of this conclusion is reduced by these limitations, and further studies with larger sample sizes and longer follow-up periods are needed.

Although statistical heterogeneity was generally low, moderate heterogeneity was observed for marginal integrity at 12 months (I^2^ = 35%). According to GRADE guidance, this level of inconsistency was not considered serious, as the direction of effects was consistent across studies and did not meaningfully affect the pooled estimates. Nevertheless, this heterogeneity may be partly explained by clinical and methodological differences among the included trials, such as variations in adhesive systems, cavity classes (Class I versus Class II), isolation methods (rubber dam versus cotton rolls), and operator-related factors. These sources of variability should be considered when interpreting the meta-analytic findings.

Bulk-fill composites demonstrated high survival rates (above 95%) in primary molars, with low rates of failure related to marginal integrity, marginal discoloration, or secondary caries. These results suggest that bulk-fill composites may be considered a reliable restorative option for primary teeth [17,21]. While GIC has therapeutic benefits such as fluoride release, our review focused primarily on bulk-fill composites and their performance in primary teeth, where they showed good mechanical properties and clinical durability [17,23]. Thus, although GIC offers therapeutic benefits, bulk-fill composites show promising mechanical performance and aesthetic maintenance, potentially offering a viable alternative for restorations in children, though further studies with longer follow-up are needed to confirm their long-term efficacy.

Bulk-fill composites performed well in both Class I and Class II cavities in primary teeth, confirming their versatility in pediatric dentistry [1,23]. Unlike previous systematic reviews [23,24], which primarily focused on short-term outcomes and a limited number of studies, our review includes a larger pool of recent randomized controlled trials (RCTs) with longer follow-up periods. This allows for a more comprehensive evaluation of the long-term survival rates and performance of bulk-fill composites in primary teeth. Additionally, we assessed a broader range of outcomes, including secondary caries incidence and color stability, which were not fully explored in earlier syntheses. Our methodology also includes the use of the GRADE approach to assess the quality of the evidence, providing a clearer understanding of the reliability of the results compared to previous reviews.

Evaluating the performance of restorative materials in both cavity types is therefore essential to determine their applicability and durability in pediatric patients. In a two-year follow-up, Sarapultseva et al. (2019) [19] observed similar performance between flowable bulk-fill composites and nanoceramic composites in Class I cavities, with no statistically significant differences in success rates. Comparable results were reported by Ehlers et al. (2019) [18] in Class II cavities, with equivalent functional performance between bulk-fill and conventional composites, although the latter showed a slight aesthetic advantage. These findings suggest that bulk-fill composites appear to be clinically safe in primary teeth, maintaining marginal integrity and anatomical stability in follow-ups of up to 12 months.

Marginal adaptation is critical to the longevity of restorations in primary teeth, and bulk-fill composites performed well in maintaining marginal integrity, particularly in Class II cavities [17,25]. The viscosity of the resin directly influences marginal adaptation, with high-viscosity composites showing better sealing compared to flowable ones, while the insertion technique—bulk-fill versus incremental—can impact gap formation, with the bulk-fill technique being effective when properly applied [26].

The use of universal adhesives, appropriate light-curing protocols, and rubber dam isolation is key to optimizing marginal sealing and reducing failure risks. Most studies employed universal adhesives in self-etch or selective-etch modes, reflecting a simplification trend in pediatric protocols. Self-etch two-step systems like Clearfil SE Bond and Prime and Bond NT were also evaluated, showing stable performance, particularly in deeper cavities where dentin preservation and reduced technique sensitivity are needed. Both universal and multi-step systems can be effective when properly applied, with selective enamel etching potentially enhancing marginal sealing.

The use of universal adhesives, light-curing protocols, and rubber dam isolation is key to optimizing marginal sealing and reducing failure risks. Most studies employed universal adhesives in self-etch or selective-etch modes, reflecting a simplification trend in pediatric protocols. Modern polywave LED curing units with high irradiance likely contributed to the successful polymerization of bulk-fill composites, reinforcing their suitability in pediatric dentistry. The bulk-fill technique demonstrates clinical performance similar to the incremental method, with annual failure rates between 1% and 1.2% [18].

From a technical perspective, bulk-fill composites offer significant advantages, mainly due to simplification of the restorative technique [21]. The ability to apply thicker increments reduces the number of clinical steps, decreases cavity exposure time and risk of contamination, and increases patient comfort [4]. These aspects are particularly relevant in pediatric dentistry, where clinical time and patient cooperation may be limited [2]. The results of this systematic review indicate that bulk-fill composites have clinical performance comparable to conventional composites in posterior restorations of primary teeth, with high survival rates and low occurrences of failures related to marginal integrity, secondary caries, or fractures in follow-ups of up to 12 months [6,7]. This consistent performance can be attributed to advances in resin formulation, such as new photoinitiators and modified fillers, which ensure adequate polymerization depth and mechanical properties even in increments of up to 4 mm. Furthermore, reducing the number of increments decreases the risk of bubble formation or contamination between layers, potentially improving marginal adaptation and restoration stability.

Despite positive results, several limitations should be considered. Firstly, the follow-up period was relatively short (6 to 24 months), which limits the ability to assess the long-term performance of bulk-fill composites in primary teeth. Additionally, there was methodological heterogeneity across the included studies, including variations in adhesive systems, cavity types, and isolation techniques. Another limitation is the small sample size in some studies, which may have impacted the statistical power of the analyses.

Additionally, the potential impact of publication bias was not fully addressed. The decision to restrict the inclusion to RCTs may have excluded valuable evidence from non-randomized studies, which could provide complementary insights. Furthermore, excluding studies based on their statistical models could have influenced the results, as some studies may have used different methodologies that could offer additional perspectives on the outcomes.

The certainty of evidence for the evaluated clinical outcomes was classified as moderate according to the GRADE approach. Three randomized clinical trials contributed to each outcome (marginal discoloration, marginal integrity, and color match). Although the risk of bias, inconsistency, and indirectness were not considered serious, imprecision related to the limited number of trials, small sample sizes, and incomplete reporting of confidence intervals led to downgrading the certainty of evidence. Some concerns related to blinding and losses to follow-up across studies further contributed to this imprecision, resulting in a moderate level of certainty. Statistical heterogeneity was low for color match and marginal discoloration (I^2^ = 0%) and moderate for marginal integrity (I^2^ = 35%).

In addition, the restriction of the meta-analyses to the worst clinical scores may have underestimated moderate differences between materials, particularly for outcomes presenting intermediate changes that did not reach failure thresholds. Quantitative synthesis was limited to baseline/short-term and 12-month follow-up periods, as longer follow-up data (18 and 24 months) were inconsistently reported across studies or available in a limited number of trials, precluding reliable quantitative pooling.

In addition, considerable methodological variability was observed among the included studies, which should be taken into account when interpreting the pooled results. Differences in cavity classifications and cavity size, adhesive systems and application protocols, light-curing devices and irradiation parameters, as well as operator experience and calibration, may have directly influenced restoration performance and failure rates. Furthermore, variability in randomization procedures, allocation concealment, and blinding of operators and outcome assessors may have introduced performance and detection bias in some trials. These methodological differences, together with variations in follow-up duration, limit the comparability of the included studies and may affect the generalizability of the findings to different clinical settings in pediatric dentistry.

Publication bias could not be formally assessed due to the limited number of randomized clinical trials included per outcome, which precludes reliable interpretation of funnel plots or statistical tests. Although trial registries and gray literature sources were searched, no additional eligible studies were identified. Therefore, a potential risk of publication bias cannot be completely ruled out. Despite these limitations, the available evidence indicates that bulk-fill composites show clinical performance comparable to conventional composites and glass ionomer cements in primary molars.

From a clinical perspective, the results indicate that bulk-fill composites may represent an effective and safe alternative for restorations in primary teeth, providing reduced operative time and simplified application without compromising restoration quality or longevity. Future studies should prioritize multicenter randomized clinical trials with rigorous methodological standardization, long-term follow-up, and comprehensive assessment of clinical outcomes, including pulpal health, structural integrity, aesthetic performance, and incidence of secondary complications. Additionally, the development and evaluation of bioactive materials capable of releasing calcium, phosphate, and fluoride ions, promoting remineralization and preventing secondary caries, are recommended. In the context of pediatric dentistry, investigations incorporating standardized measures of patient acceptance, behavior during treatment, and caregiver satisfaction could provide additional valuable insights for clinical practice, contributing to evidence-based decisions and more effective management strategies.

## 5. Conclusions

Bulk-fill resin composites demonstrate favorable clinical performance in the restoration of primary molars in pediatric patients, with survival rates comparable to those of conventional resin composites over follow-ups of up to 12 months, although data from 18 to 24 months are also available in some studies. Failure rates related to marginal integrity, secondary caries, and fractures were generally low across the included studies. Although most trials presented a moderate risk of bias, the overall certainty of evidence was rated as moderate, mainly due to limitations related to sample size, imprecision, and the lack of long-term data. Within these constraints, the available evidence suggests that bulk-fill composites represent a reliable and efficient restorative option for primary dentition, offering predictable short- to medium-term clinical outcomes, but further studies are needed to confirm long-term performance.

## Figures and Tables

**Table 1:** Electronic bibliometric search strategies.

Database	Search Strategy
Pubmed	(Bulk Fill) OR (Bulkfill) OR (Bulk-fill) OR (Bulk Fill) OR (Bulk Filled) OR (Bulk Filling) AND (Restorations, Permanent Dental) OR (Restoration, Permanent Dental) OR (Dental Permanent Fillings) OR (Dental Permanent Filling) AND (Primary teeth) OR (deciduous tooth) OR (primary tooth) OR (deciduous teeth) OR (primary dentition) OR (primary dentitions) OR (milk teeth) OR (milk tooth) OR (deciduous dentition) OR (deciduous dentitions)
Embase	bulkfill OR ‘bulk fill’ OR ((‘bulk’/exp OR bulk) AND fill) OR ‘bulk filled’ OR ((‘bulk’/exp OR bulk) AND filled) OR ‘bulk filling’ OR ((‘bulk’/exp OR bulk) AND filling) AND ‘restorations, permanent dental’ OR (restorations, AND permanent AND (‘dental’/exp OR dental)) OR ‘restoration, permanent dental’ OR (restoration, AND permanent AND (‘dental’/exp OR dental)) OR ‘dental permanent fillings’ OR ((‘dental’/exp OR dental) AND permanent AND fillings) OR ‘dental permanent filling’ OR ((‘dental’/exp OR dental) AND permanent AND filling) AND ‘primary teeth’/exp OR ‘primary teeth’ OR (primary AND (‘teeth’/exp OR teeth)) OR ‘deciduous tooth’/exp OR ‘deciduous tooth’ OR (deciduous AND (‘tooth’/exp OR tooth)) OR ‘primary tooth’/exp OR ‘primary tooth’ OR (primary AND (‘tooth’/exp OR tooth)) OR ‘deciduous teeth’/exp OR ‘deciduous teeth’ OR (deciduous AND (‘teeth’/exp OR teeth)) OR ‘primary dentition’/exp OR ‘primary dentition’ OR (primary AND (‘dentition’/exp OR dentition)) OR ‘primary dentitions’ OR (primary AND dentitions) OR ‘milk teeth’/exp OR ‘milk teeth’ OR ((‘milk’/exp OR milk) AND (‘teeth’/exp OR teeth)) OR ‘milk tooth’/exp OR ‘milk tooth’ OR ((‘milk’/exp OR milk) AND (‘tooth’/exp OR tooth)) OR ‘deciduous dentition’/exp OR ‘deciduous dentition’ OR (deciduous AND (‘dentition’/exp OR dentition)) OR ‘deciduous dentitions’ OR (deciduous AND dentitions)
Web of Science	TS = ((Bulk Fill) OR (Bulkfill) OR (Bulk-fill) OR (Bulk Fill) OR (Bulk Filled) OR (Bulk Filling)) AND TS = ((Restorations, Permanent Dental) OR (Restoration, Permanent Dental) OR (Dental Permanent Fillings) OR (Dental Permanent Filling)) AND TS = ((Primary teeth) OR (deciduous tooth) OR (primary tooth) OR (deciduous teeth) OR (primary dentition) OR (primary dentitions) OR (milk teeth) OR (milk tooth) OR (deciduous dentition) OR (deciduous dentitions))
The Cochrane Library	(Bulk Fill) OR (Bulkfill) OR (Bulk-fill) OR (Bulk Fill) OR (Bulk Filled) OR (Bulk Filling) AND (Restorations, Permanent Dental) OR (Restoration, Permanent Dental) OR (Dental Permanent Fillings) OR (Dental Permanent Filling) AND (Primary teeth) OR (deciduous tooth) OR (primary tooth) OR (deciduous teeth) OR (primary dentition) OR (primary dentitions) OR (milk teeth) OR (milk tooth) OR (deciduous dentition) OR (deciduous dentitions)
Scopus	(TITLE-ABS-KEY ((Bulk Fill) OR (Bulkfill) OR (Bulk-fill) OR (Bulk Fill) OR (Bulk Filled) OR (Bulk Filling))) AND (TITLE-ABS-KEY ((Restorations, Permanent Dental) OR (Restoration, Permanent Dental) OR (Dental Permanent Fillings) OR (Dental Permanent Filling))) AND (TITLE-ABS-KEY ((Primary teeth) OR (deciduous tooth) OR (primary tooth) OR (deciduous teeth) OR (primary dentition) OR (primary dentitions) OR (milk teeth) OR (milk tooth) OR (deciduous dentition) OR (deciduous dentitions)))

**Table 2:** Characteristics of included studies.

Author/Year	Study Design	Volunteers (mean age/years)	Number of Restored Teeth	Isolation	Depth of Carious Lesions (mean/mm)	Follow-Ups (months)	Dental Materials	Type of Cavity	Adhesive System	Light Curing Unit	Analysis Criteria
Akman and Tosun (2020) [17]	RCT	30 (6–10)	160	Cotton rolls and suction	NR	0, 3, 6, 12	Equia (GIC) Sonic Fill (Bulk-Fill) X-tra fil (Bulk-Fill) Z350 xt	Class II	Clearfil SE Bond	Valo LED	USPHS
Ehlers et al. (2019) [18]	RCT	32 (6.7 ± 1.2)	100	NR	NR	0, 12	Dyract eXtra (control) Venus Bulk-Fill Flow	Class II	Scotchbond Universal	Elipar S 10	FDI
Saraputltseva and Sarapultseva (2019) [19]	RCT	27 (3–6)	54	NR	Maximum of 4 mm	0, 6, 18, 24	Ceram x (control) SDR Bulk-Fill Flow	Class I	Prime and Bond NT	NR	USPHS
Oter, Deniz and çehreli (2019) [20]	RCT	80 (7.41 ± 1.80)	160	Cotton rolls and suction	3, 1	0, 6, 12	Filtek Z250 (control) Filtek Bulk-Fill restorative	Class I	Single Bond Universal Adhesive	Elipar Freelight 2	USPHS
Gindri et al. (2022) [21]	RCT	65 (6.7 + 1.5)	140	Rubber dam isolation	NR	0, 6, 12	Filtek Z350 XT (control) Filtek Bulk-Fill	Class II	Scotchbond Universal	QHL 75 Curing light	FDI
Massa et al. (2022) [22]	RCT	62 (5.9 ± 1.7)	144	Rubber dam isolation	NR	18	Vitremer (GIC) Filtek Bulk-Fill	Class II Class I	Scotchbond Universal	XL 2500	FDI

NR: not reported.

**Table 3:** Clinical performance of restorative materials in primary molars according to follow-up.

Author, Year	Follow-Up	Incremental RBC	GIC	Bulk-Fill RBC
Akman and Tosun (2020) [17]	3–6 months	No failures observed (0/32)	No failures observed (0/34)	No failures observed (0/68)
	12 months	No failures observed (0/32)	Anatomical form: (01/34)	No failures observed (0/68)
Sarapultseva and Sarapultseva (2019) [19]	6–18 months	No failures observed (0/27)	NR	No failures observed (0/27)
	24 months	No failures observed (0/24)	NR	No failures observed (0/24)
Oter, Deniz and çehreli (2019) [20]	6 months	No failures observed (0/80)	NR	Retention loss: (01/80)
	12 months	Retention loss: (01/80)	NR	Retention loss: (01/80)
Gindri et al. (2022) [21]	6 months	Marginal integrity: (02/70) Fracture: (01/70)	NR	Marginal integrity: (03/70) Fracture: (01/70)
	12 months	Marginal integrity: (02/70) Secondary caries: (01/70) Fracture: (02/70)	NR	Marginal integrity: (05/70 Secondary caries: (01/70) Fracture: (01/70)
Massa et al. (2022) [22]	18 months	NR	Marginal discoloration: (05/72) Secondary caries: (04/72) Fracture: (07/72) Anatomical form: (04/72)	Marginal discoloration: (02/72) Marginal integrity: (05/72) Secondary caries: (02/72) Fracture: (10/72) Anatomical form: (02/72)

NR: indicates that the material was not evaluated in that study.

**Table 4:** Quality of the evidence: GRADE assessment.

Certainty Assessment									
Parameter	No of Studies	Study Design	Risk of Bias	Inconsistency	Indirectness	Imprecision	Other Considerations	Important	Certainty
Marginal Discoloration	3	RCT	Not serious ^a^	Not Serious ^b^	Not Serious ^c^	Serious ^d^	None		⊕⊕⊕O Moderate
Marginal Integrity	3	RCT	Not serious ^a^	Not serious ^b^	Not Serious ^c^	Serious ^d^	None		⊕⊕⊕O Moderate
Color Match	3	RCT	Not serious ^a^	Not serious ^b^	Not Serious ^c^	Serious ^d^	None		⊕⊕⊕O Moderate

GRADE Working Group grades of evidence. ⊕⊕⊕⊕ High quality: Further research is very unlikely to change our confidence in the estimate of effect. ⊕⊕⊕O Moderate quality: Further research is likely to have an important impact on our confidence in the estimate of effect and may change the estimate. ⊕⊕OO Low quality: Further research is very likely to have an important impact on our confidence in the estimate of effect and is likely to change the estimate. ⊕OOO Very low quality: We are very uncertain about the estimate. ^a^ Risk of bias was judged as not serious at the individual study level; however, some concerns related to incomplete reporting of blinding and losses to follow-up across studies led to downgrading the certainty of evidence to moderate. ^b^ No substantial heterogeneity was identified across studies; results were consistent. ^c^ Indirectness was assessed considering population, intervention, comparison, and outcomes. The studies used pediatric samples with primary teeth and clinical outcomes directly applicable to practice; therefore, they were not downgraded. ^d^ The evidence was downgraded for imprecision due to small sample sizes and lack of clear confidence intervals, but was not downgraded for other domains.

## Data Availability

All data supporting the findings of this systematic review are available within the manuscript. In addition, coded data extraction forms and supplementary datasets generated during the review process are available from the corresponding author upon reasonable request.

## Supplementary Materials

The following supporting information can be downloaded at https://www.mdpi.com/article/10.3390/jcm15020415/s1. Table S1: PRISMA 2020 Checklist. Table S2: Full-text studies excluded after eligibility assessment and reasons for exclusion.
