# Supplementary material for: Clinical Performance and Survival of Bulk-Fill Resin Composite Posterior Restorations in Primary Teeth: A Systematic Review and Meta-Analysis
Source: J Clin Med. 2026 Jan 6;15(2):415. doi: 10.3390/jcm15020415 (PMC12842217; doi:10.3390/jcm15020415)
Supplement: Supplementary file 1 [file jcm-15-00415-s001.zip › Table S2.pdf]

Supplementary Table S2. Full-text studies excluded after eligibility assessment and reasons for exclusion.

Author(s), Year	Title	Reason for exclusion
Olegário et al., 2016	Effectiveness of conventional treatment using bulk-fill composite resin versus atraumatic restorative treatment in primary and permanent dentition: A pragmatic randomized clinical trial	Mixed dentition (primary and permanent teeth), with outcomes not reported separately for primary teeth
Lardani et al., 2022	One-year clinical performance of Activa™ Bioactive-Restorative Composite in primary molars	Use of Activa™ BioActive as comparator, which was not included among the predefined comparators of this review
Uzel et al., 2022	Evaluation of glass-ionomer versus bulk-fill resin composite: A two-year randomized clinical study	Evaluated permanent teeth only
Atabek et al., 2017	Two-year clinical performance of a sonic-resin placement system in posterior restorations	Evaluated permanent posterior restorations
Olegário et al., 2022	Use of rubber dam versus cotton roll isolation on composite resin restorations' survival in primary molars: Two-year results from a non-inferiority clinical trial	Focused on isolation techniques; outcomes not compatible with the predefined clinical endpoints and effect measures of this review
